# Rapid accumulation of fluorophores and fast kill identify drugs with bactericidal effects against Gram-negative bacteria

**DOI:** 10.1099/mic.0.001619

**Published:** 2025-11-13

**Authors:** J. Enrique Salcedo-Sora, Douglas Bruce Kell

**Affiliations:** 1 GeneMill Research Facility, Liverpool Shared Research Facilities, University of Liverpool, Crown Street, Liverpool, L69 7ZB, UK; 2 Department of Biochemistry Cell and Systems Biology, Institute of Systems, Molecular and Integrated Biology, University of Liverpool, Crown Street, Liverpool, L69 7ZB, UK; 3 The Novo Nordisk Foundation Center for Biosustainability, Danish Technical University, Søltofts Plads 200, 2800 Kgs. Lyngby, Denmark

**Keywords:** antibiotics, *A. baylyi*, cell membrane, *E. coli*, flow cytometry, fluorophores, Gram-negative, polymyxins, Prestwick library

## Abstract

Antimicrobial resistance is a massive threat, but developing a new antibiotic can take decades. That time could be drastically reduced if we were able to anticipate desirable properties of a chemical, such as its potential to target specific bacterial compartments. This would provide the opportunity to prioritize the development of molecules that target, for instance, the cell membrane, as this does not rely on transporters and usually results in a fast-acting bactericidal effect. We used flow cytometry and a set of fluorophores together with a group of antibiotics to discriminate between antimicrobials acting on cell membrane versus intracellularly against two Gram-negative bacteria, *Escherichia coli* and *Acinetobacter baylyi*. We then chose Rhodamine 123 as a fluorescent marker to screen a commercial library of chemical compounds. Using flow cytometry, several drugs present in the Prestwick library were observed to have cytotoxic effects at 1 µM final concentration towards *E. coli*. This was confirmed with growth inhibitory assays in both *E. coli* and *A. baylyi* for pantoprazole, theophylline and zoledronic acid. This represents an approach to the large-scale screening of small molecules with the potential to deliver fast-acting molecules that target cell membranes in Gram-negative bacteria.

**Impact Statement**

The discovery of novel antimicrobials is essential to build resilience against infectious diseases. Understanding the mechanism of action of known as well as novel compounds is equally crucial. Achieving this understanding is a long and complex process that is usually addressed on an individual basis for an antibiotic, or a class of antibiotics. Using high-throughput pipelines such as those possible with flow cytometry can help to shorten this process. We present a flow cytometry-based approach capable of determining if a given molecule has rapid antimicrobial effects. The same approach measured the increase in the permeability of the bacterial cell membrane. The latter property in particular usually makes a chemical a very effective antimicrobial.

## Data Summary

The authors confirm that all supporting data, code and protocols have been provided within the article or through supplementary data files. Supplementary material is available through Figshare at 10.6084/m9.figshare.30112513 [1]

## Introduction

The membrane transport of natural substrates as well as xenobiotics in live cells is mediated by proteins embedded in the otherwise impermeable cell membrane [2,3]. Accordingly, antibiotics targeting the bacterial cell membrane, which make for a formidable barrier, are very effective bactericides [4]. Molecules that preferentially target the cell membrane are much sought after, with the majority of antibiotics in preclinical and clinical development seeking to target cell wall and membrane synthesis [5,7]. However, it still commonly takes a further 10 years for the first of any of the successful drugs to reach the market [5,7].

Flow cytometry can interrogate a cell’s physiology rapidly and on an individual-cell basis. This technology has prospered throughout microbiological research ever since the technology was acknowledged to be applicable to the much smaller microbial cells [8,13]. More recently, flow cytometry has been utilized for the study of membrane transport [14]. With the wealth of fluorogenic dyes under continuous expansion, flow cytometry has allowed us to interrogate and compare, for instance, influx versus efflux membrane transport systems [15]. The use of fluorogenic compounds as a proxy for transporter substrates has benefited from the structural similarities that often exist between available fluorophores and transporter substrates [16]. This has facilitated the comparative measurement of transport of xenobiotics by specific membrane proteins [17,18].

We leveraged these developments to assemble a screening method that detects lethal cell wall and cell membrane-interacting compounds. Conventional antibiotic sensitization screens often capture large numbers of hit molecules with unrelated activities that will require further characterization. Given the multiparametric nature of flow cytometry, while detecting cell accumulation of fluorogenic dyes [19], we exploited here the capacity of a palette of dyes to reveal cell damage in response to a short exposure to a range of known antibiotics in *Escherichia coli* and *Acinetobacter baylyi*. These antibiotics represented examples of antibacterials considered to affect either the cell membranes or intracellular targets. The most discriminatory parameters derived from these assays were then applied to interrogate the Prestwick chemical library of marketed drugs to identify novel antimicrobial candidates. The bactericidal effects found in pantoprazole, theophylline and zoledronic acid were then confirmed against both Gram-negative bacteria, *E. coli* and *A. baylyi* by standard growth inhibitory assays.

## Methods

### Bacterial strains and chemicals

Strains: *E. coli* BW25113 [*Δ(araD-araB)567*, *ΔlacZ4787(::rrnB-3)*, *λ −*, *rph-1*, *Δ(rhaD-rhaB)568, hsdR514*] and *A. baylyi* ADP1 (ATCC 33305). Antibiotics were purchased from Merck KGaA (Darmstadt, Germany) and prepared in PBS (50 mg ml^−1^ stocks, filter-sterilized), except for nitrofurantoin, pentamidine and rifampicin which needed DMSO to dissolve, and chloramphenicol and tetracycline that were dissolved in ethanol. Antibiotic stocks were stored at −20 °C in small volume aliquots and disposed of after three rounds of freezing and thawing.

### Growth and permeability effects measured by flow cytometry

Fluorophores were purchased from Sigma-Aldrich (Merck KGaA), ThermoFisher, TCI America or ATTO-TEC GmbH. Other reagents were purchased from Sigma-Aldrich. Fluorophore uptake assays started with the spread of a small flake from a frozen glycerol stock of bacteria onto a fresh plate of complex solid media (Merck LB 110283) and incubated overnight at 37 °C. A single colony from a fresh solid media plate was then incubated in 5 ml of complex media (Merck LB 110285) overnight at 37 °C with shaking at 250 r.p.m. in the absence of antibiotics. The overnight cultures were diluted 1: 1,000 in fresh liquid complex media and grown for 2 h at 37 °C with shaking at 250 r.p.m. The cell density was then adjusted to ~2,000 cells per microlitre, as inferred by turbidity (OD_600_=0.3). For antibiotic exposure, cells were first incubated in complex media for 30 min, 37 °C, in 384-well plates, at a final volume of 50 µl. Antibiotics were present at final concentrations equivalent to twice their IC_50_ values for either *E. coli* or *A. baylyi*. We use 2× IC_50_ to approach the concentrations with maximal efficacy for a given compound [20]. Fluorophores were then added at 1 µM final concentration, and cells were incubated for a further 10 min at 37 °C (similar to our previous report [15]) before interrogation in the flow cytometry analyser as detailed below.

### Flow cytometry

We used a high-throughput flow cytometer, the Intellicyt iQue Screener Plus (Sartorius, Göttingen, Germany), with the following protocol: buffer equilibration (QSol, Sartorius) and plate shaking 2,000 r.p.m. for 50 s, sampling for 2 s with 1 s upload time, 5 s wash in Qsol buffer every three wells and further probe wash for 10 s every 12 wells. The instrument has three light-emitting diode lasers (405 nm, 488 nm, 640 nm) and collects data for 2 light-scattering channels and 13 fluorescence channels. Once the light from any of three lasers has reached the samples, these channels collect the fluorescent (emission) signals back from the samples in the following spectral ranges (channel name, emission range in nm): VL1 (445±45), VL2 (530±30), VL3 (572±28), VL4 (615±24), VL5 (675±30), VL6 (780±60), BL1 (530±30), BL2 (572±28), BL3 (615±24), BL4 (675±30), BL5 (780±60), RL1 (675±30) and RL2 (780±60). Initial gating used forward versus side scatter to select the region with cells of similar size and granularity. Singlet gating was applied using height versus width of FSC. Data collected for downstream analysis consisted of the number of events (single cells) and the median values of the fluorescent signal. This instrument is highly resistant to the detection of extracellular fluorescence, a potential source of noise in fluorescent assays [21]. As with any other transport or cell accumulation assays (e.g. filtration or flow dialysis [22]), flow cytometry would not discriminate directly between molecules that bind outward-facing cell structures from those molecules that have actually reached intracellular compartments.

### Screening of chemical library

The Prestwick Chemical Library® of mostly FDA-approved small molecules was used to test for antibacterial activity of non-antibiotic drugs against *E. coli*. A set of 1,280 compounds at 10 mM in DMSO (full list in Table S1, available in the online Supplementary Material) was aliquoted out in 384-multi-well plates for testing at final 1 µM concentration in PBS (0.01% v/v DMSO final concentration) using in-house robotics for liquid handling [GeneMill Research Facility, Liverpool Shared Research Facilities (LIV-SRF), University of Liverpool]. The negative control was 1% v/v DMSO and the positive control was colistin (3 mg l^−1^). Bacterial cultures growing in logarithmic phase in complex media were added to 50 µl final volume per well. After a 30-min incubation at 37 °C, Rhodamine 123 (a dye normally excluded by Gram-negative bacteria) [23] was added to 1 µM final concentration for a further incubation of 15 min at 37 °C.

### Data analysis

End-point growth inhibitory concentrations were calculated from microtitration assays. The inhibitory concentrations of antibiotics that kill half of the bacterial population (IC_50_) were calculated with the four-parameter logistic model as implemented in py50 [24]. Flow cytometry data were analysed using a combination of the instrument’s Forecyt software and routines written by the first author in R and Python. The principal component analyses were carried out using R’s packages FactoMineR [25].

### Inhibitory concentration assays

Bacterial cultures were carried out in complex media (lysogeny broth, Merck LB 110285). Overnight cultures in complex media were diluted 1 : 1,000 in fresh LB and incubated for 2 h at 37 °C, shaking 250 r.p.m. One hundred microlitres of twofold dilutions of drugs were prepared in flat-bottom 96-well plates to which 100 µl of fresh bacterial culture was added. Controls included the absence of antibiotic (negative control) as well as colistin 3 mg l^−1^ (positive control). Endpoint read-outs of the media turbidity at 600 nm (OD_600_) were taken after an overnight incubation (~20 h) at 37 °C, using a BMG LabTech CLARIOstar Plus plate reader.

## Results

### Testing the sensitivity of *E. coli* and *A. baylyi* strains to a panel of antibiotics

We tested 19 antibiotics in current clinical use (Table 1). Eight antibiotics primarily targeting the cell membrane were included: ampicillin, amoxicillin, bacitracin, colistin (polymyxin E), fosfomycin, pentamidine, polymyxin B and vancomycin. The rest of the antibiotics in Table 1 were considered to have primary targets that are intracellular. The sensitivity of both *E. coli* BW25113 and *A. baylyi* ADP1 to this set of antibiotics was measured as growth inhibition in microtiter plates after 20-h incubation (Table 1). These values informed the later use of this set of antibiotics in the present work.

**Table 1.** Inhibitory concentrations (IC_50_) for *E. coli* and *A. baylyi* The average of at least three assays (mean) and the sd are listed. The first eight antibiotics (shaded) are categorized as cell membrane acting. The others are categorized as intracellularly acting antibiotics.

	*E. coli*		*A. baylyi*	
	Mean (mg l^−1^)	sd	Mean (mg l^−1^)	sd
Ampicillin	1.48	0.32	23.4	4.57
Amoxicillin	1.05	0.95	0.43	0.40
Bacitracin	>1,000	na	48.4	2.56
Colistin	0.18	0.10	0.15	0.02
Fosfomycin	0.25	0.24	15.3	14.4
Pentamidine	71.1	2.79	46.4	2.13
Polymyxin B	85.3	5.3	>500	na
Vancomycin	68.9	55.4	46.0	42.6
Azithromycin	4.95	4.67	0.11	0.11
Cefotaxime	0.01	<0.01	1.85	1.5
Chloramphenicol	0.70	0.21	0.82	0.12
Ciprofloxacin	<0.01	na	0.040	na
Gentamicin	1.29	0.06	1.11	0.12
Nalidixic acid	2.57	1.98	1.34	1.08
Nitrofurantoin	2.08	1.98	68.3	44.3
Ofloxacin	0.05	0.01	0.08	0.01
Rifampicin	0.55	0.14	0.08	0.01
Tetracycline	0.46	0.03	0.75	0.03
Trimethoprim	0.08	0.07	7.05	6.15

NA, non-applicable.

### Testing the permeability of *E. coli* and *A. baylyi* to fluorophores

**Fig. 1.**
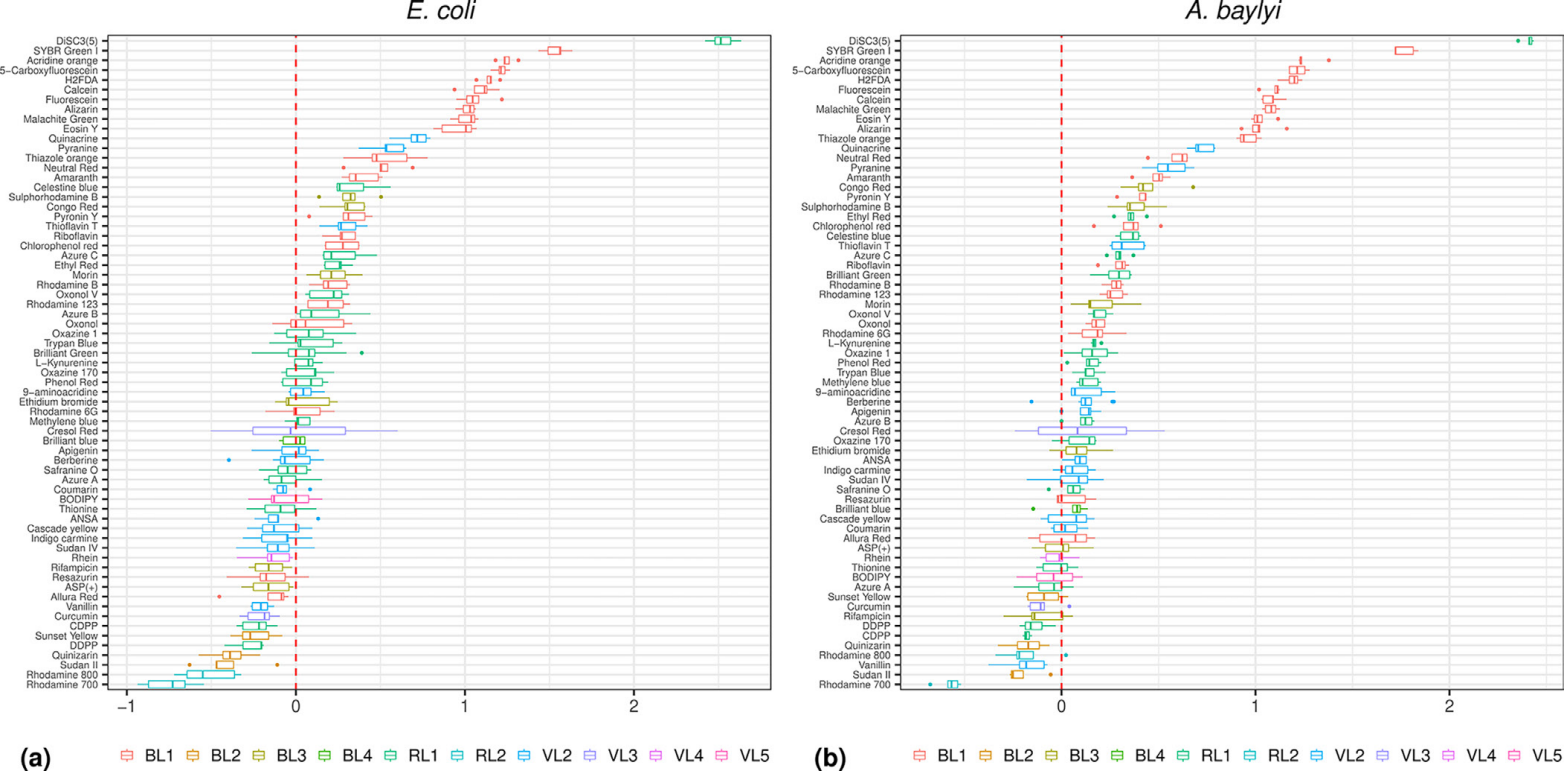
Cell accumulation of a panel of 67 fluorophores. (**a**) *E. coli*. (**b**) *A. baylyi*. Fluorophores are listed on the ordinate. The bar plots represent the distribution of the data from cellular accumulation of those fluorophores in four or more assays. The colour code of the bar plots represents the flow cytometer channels (listed below each plot) appropriate for each fluorophore in the Intellicyt iQue Screener Plus cytometer (Methods). The abscissa represents the ratio, as log_10_ values, of the fluorescence signal generated by each fluorophore against the background signal (cells in media without fluorophores). A log ratio higher than zero denotes net cellular accumulation of fluorophores. The samples with log ratios of zero or lower were assumed not to accumulate the given fluorophores.

An extensive variety of fluorogenic compounds have been previously shown to accumulate with different levels of permeability in *E. coli* [15]. This provides the opportunity to use fluorescent-based assays to interrogate the chemical space of membrane transporters that have evolved under Gram-negative bacteria [15]. We applied these fluorophores to investigate membrane permeability relevant to antibiotic uptake. The inclusion of *A. baylyi* – as a model for pathogenic *Acinetobacter* such as *Acinetobacter baumannii* – expanded the relevance of this work to other Gram-negative bacteria of current clinical importance [26]. *E. coli* and *A. baylyi* were exposed to 67 fluorophores chosen from the previous palette tested in *E. coli* [15]. The range of cell accumulation as measured by flow cytometry (Fig. 1a) corroborated the permeability profile previously reported for *E. coli* [15].

For *A. baylyi,* this is a first description of its permeability profile to a set of fluorophores. *A. baylyi* is naturally transformable [27,28] and generally had higher levels of permeability in comparison to *E. coli* (Fig. 1b). *A. baylyi* showed 52 compounds accumulating at levels greater than the background (Fig. 1b, ratios as log_10_ values equal or above 0 in the abscissa), while the corresponding value in *E. coli* was 37 compounds. This could be interpreted as some indication of the high levels of membrane permeability expected for bacteria known to be naturally competent (i.e. uninduced uptake of DNA) such as * A. baylyi* [29]. The same ten compounds were accumulated tenfold or higher in both species (Fig. 1, ratios as log_10_ values equal to or greater than 1 in the abscissa) with the cyanine DiSC3(5) exhibiting the highest accumulation: two orders of magnitude above background.

### Interactivity of fluorophore accumulation and antibiotic exposure

Twenty-three fluorophores representing the range interrogated above were selected, and their accumulation was studied in cells exposed to each of the 19 antibiotics chosen (Table 1). This was carried out against both *E. coli* and *A. baylyi*. Very apparent effects were observed on cell accumulation of fluorophores after 30-min exposure to antibiotics alone followed by 15 min of incubation with fluorophores. In *E. coli*, cell membrane-acting antibiotics such as colistin caused an increase of tenfold and above in the cellular accumulation of almost all 23 fluorophores tested, except for chlorophenol red and Congo red (Fig. S1). The data represented here are the ratios of the fluorescence signals from antibiotic-exposed cells against the fluorescence signals from non-antibiotic controls. As is usually acknowledged in cell membrane transport research, the cell accumulation of a given compound can be the result of net influx as well as the possibility of inhibited efflux [2].

**Fig. 2.**
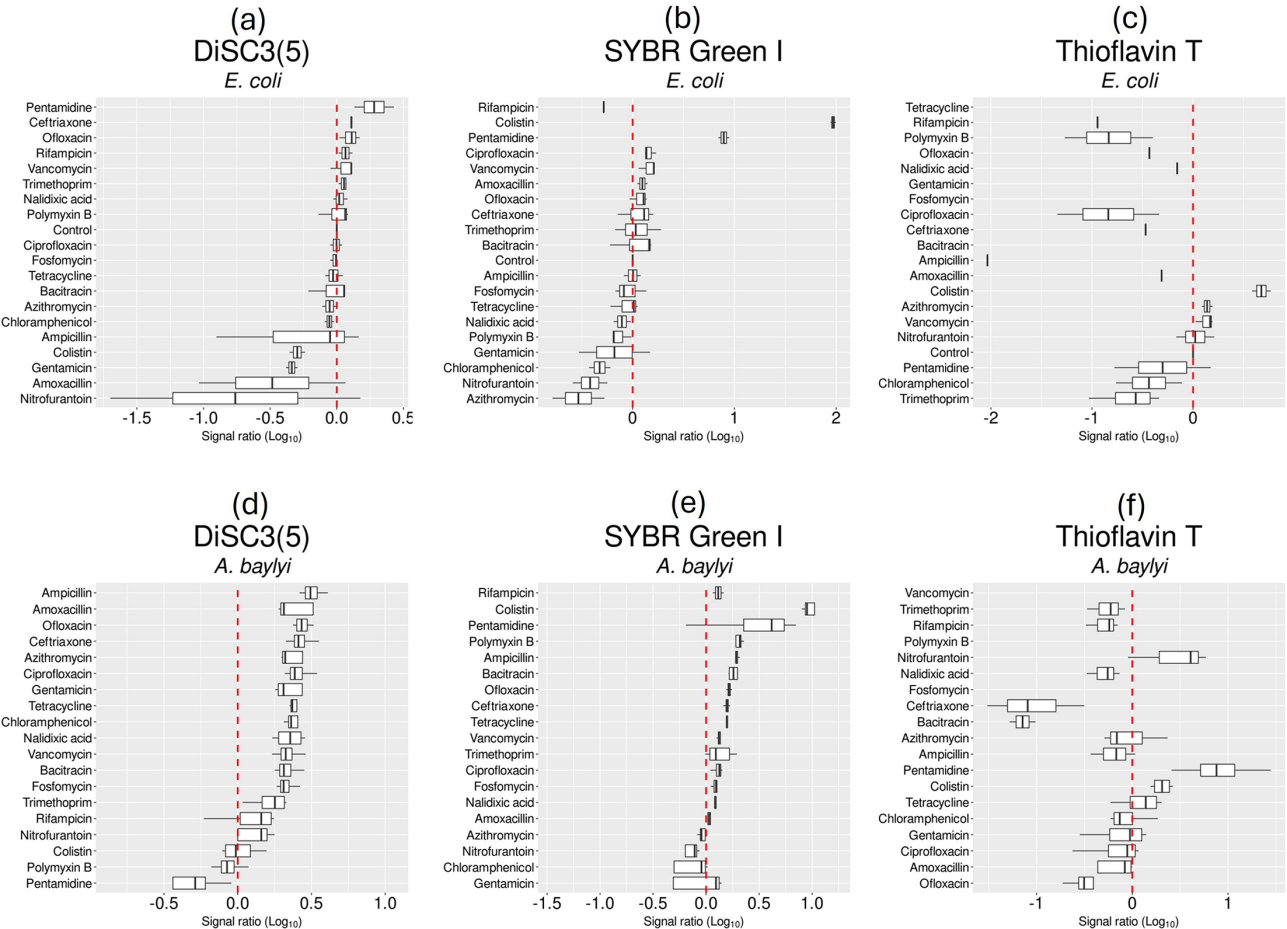
Examples of specific fluorophore uptake in *E. coli* and *A. baylyi* exposed to antibiotics. (**a–c**) *E. coli*. (**d–f**) *A. baylyi*. Fluorescence signals from three fluorophores selected from the set of 67 molecules (Fig. S1). The signals (signal ratio) for all fluorophores (Log10 values, abscissa) are shown against each antibiotic as ratios against non-antibiotic controls. The cellular accumulation of the fluorophore upon exposure to each antibiotic is represented by at least four biological replicates.

Exposure to pentamidine – another cell membrane-acting drug [30] – caused a tenfold increase in the accumulation of SYBR green, while vancomycin caused the accumulation of Allura red, amaranth and calcein (Fig. S1). In *A. baylyi*, exposure to most antibiotics increased the cell accumulation of DiSC3(5). Cell membrane inhibitors such as colistin or pentamidine caused a tenfold or higher accumulation of alizarin, Rhodamine 123 and thioflavin T (Fig. S1). These trends for DiSC3(5), SYBR green and thioflavin T illustrated the species-specific differences (Fig. 2). For instance, although most antibiotics induced higher permeability to DiSC3(5) in *A. baylyi* than in *E. coli*, colistin and pentamidine tended to stand out in causing the specific accumulation of SYBR and thioflavin T in both species (Fig. 2).

### Dimensional reduction of the multiple parameters detected in flow cytometry separates data according to antibiotic target as well as species

**Fig. 3.**
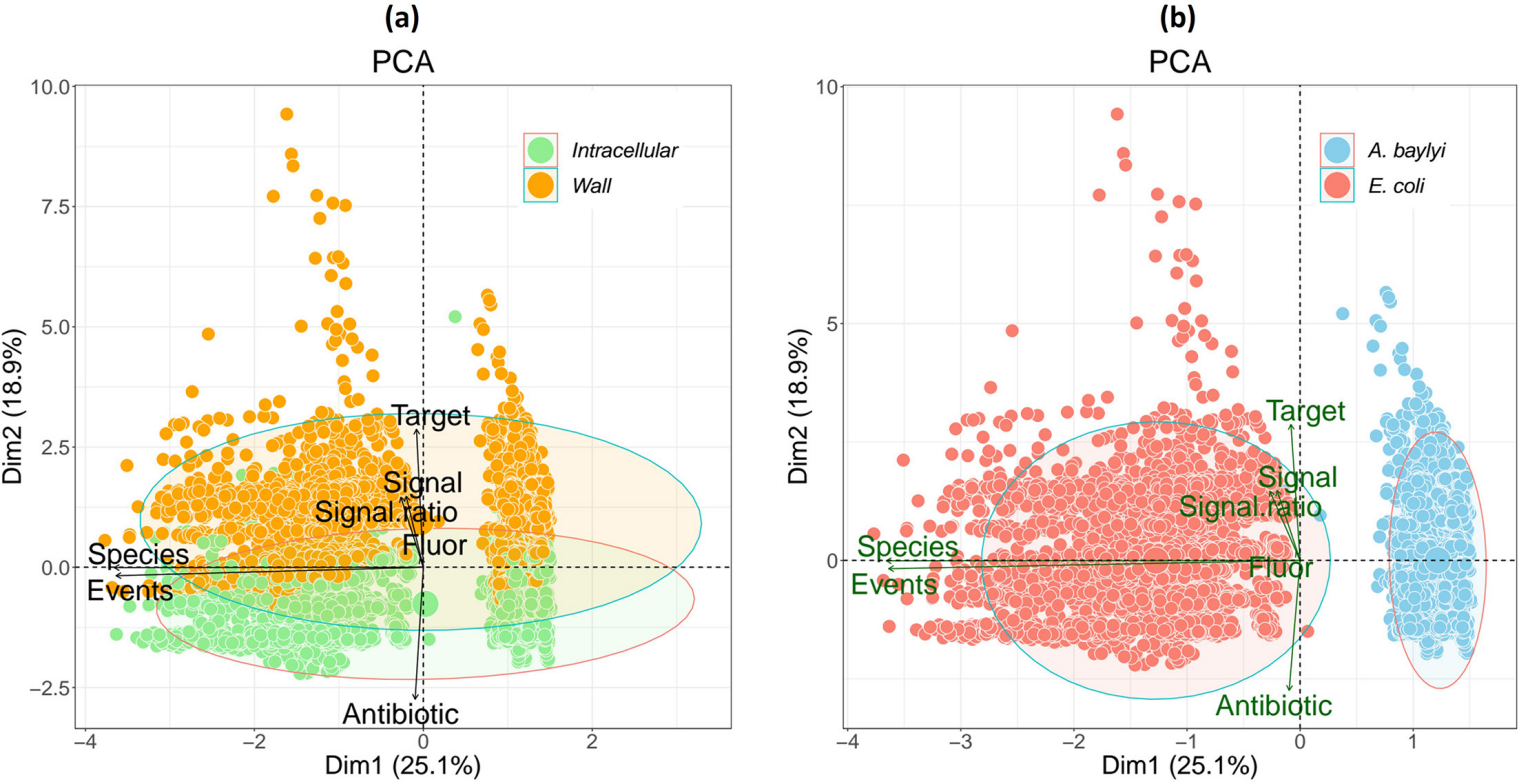
PCA of fluorophore uptake in *E. coli* and *A. baylyi* exposed to antibiotics. (**a**) Intracellular versus cell membrane-acting antibiotics. (**b**) *A. baylyi* versus *E. coli*. Fluorescence signals from 23 fluorophores (Table S2) in cells exposed to 19 different antibiotics. Data generated from four or more biological replicates. Components consisted of species (*E. coli* and *A. baylyi*), antibiotics, fluorophore, events (median), signal (median) and signal ratio (fluorescence emission from samples exposed to antibiotic versus samples without exposure to antibiotics) and target type for each antibiotic (Tables S1 and S2).

The data generated with the 23 fluorophores and 19 antibiotics were normalized and used to test for the parameters that differentiate cell membrane-acting from intracellular-acting antibiotics. The parameters used were species (*E. coli* and *A. baylyi*), antibiotic, fluor, events (median of cell count by cytometry), fluorescence signal (median of the emission) and fluorescence signal ratio (Table S2). When scoring the target (denoted as intracellular or wall) as the dependent variable, the principal component analysis (PCA) – despite being an unsupervised method with no knowledge of this – showed the data approximately segregating into two regions in one of the dimensions (Fig. 3a). Clear orthogonality was shown by the fluorophore signal (either as net fluorescence or as a ratio of exposed versus non-exposed to antibiotics), antibiotic and the number of events detected by flow cytometry (Fig. 3a). Number of events together with the net fluorescence signal provided an 80% cumulative explained variance threshold. Interestingly, even more apparent clusters were seen for the species, *E. coli* versus *A. baylyi* (Fig. 3b). The platform of the set of fluorophores and multiple known antibiotics, as implemented in this work, captured the differences in both cellular permeability and antibiotic sensitivity in these two Gram-negative bacteria.

### Screening of the Prestwick small molecule chemical library in *E. coli*

**Fig. 4.**
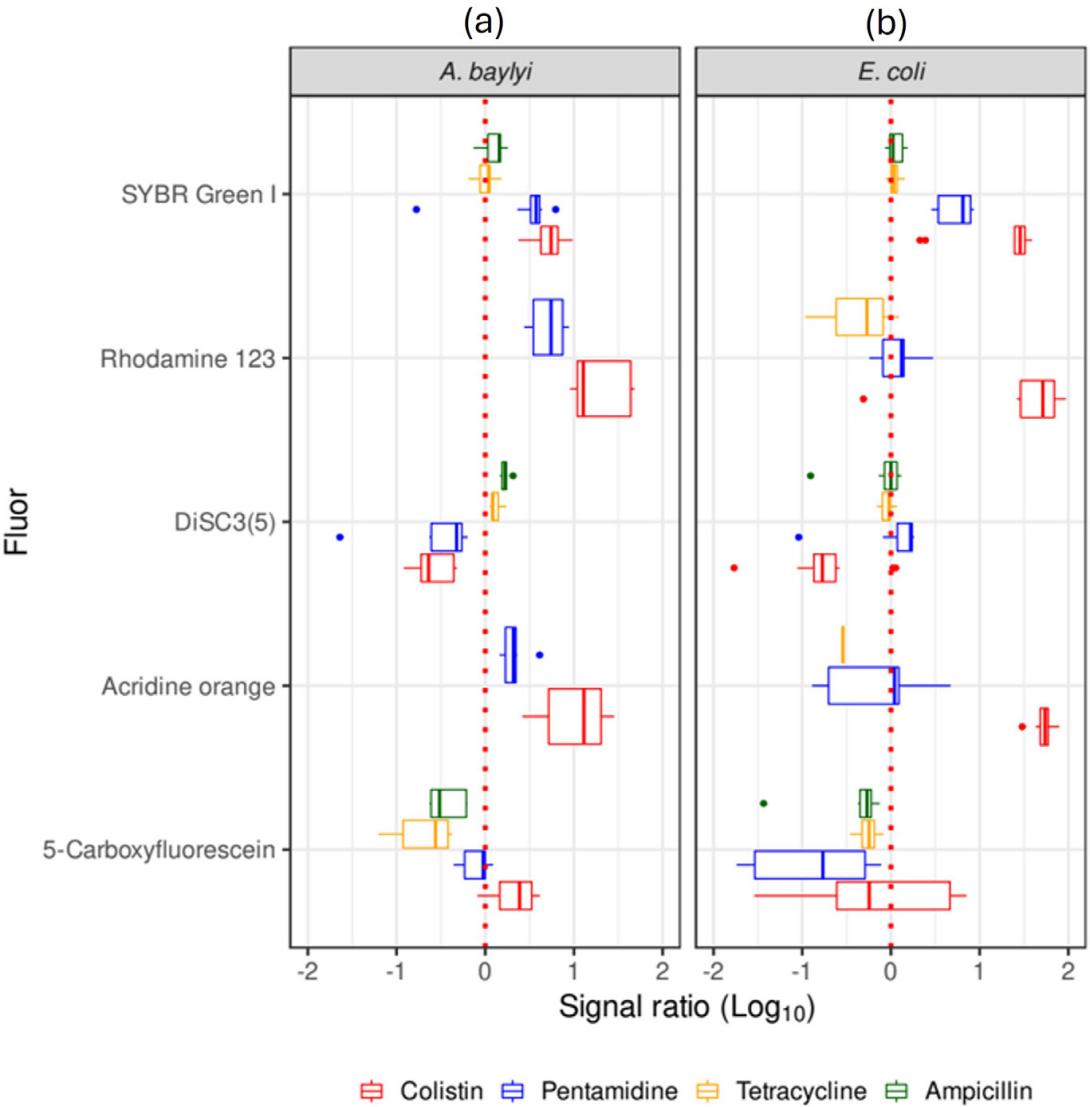
Antibiotics with the strongest effect on rapid fluorophore cell accumulation. (**a**) *A. baylyi*. (**b**) *E. coli*. Fluorophore uptake in *E. coli* and *A. baylyi* exposed to antibiotics is represented as the signal ratio of fluorescence from exposed over non-exposed to antibiotics (Log10 values, abscissa). The four antibiotics and their effect on the accumulation of each of these five fluorophores are represented as box plots: the higher the permeability caused by the exposure to a given antibiotic, the higher the signal ratio. Colour code: red, colistin; blue, pentamidine; yellow, tetracycline; green, ampicillin. The ratios for Rhodamine 123 and acridine orange post-exposure to tetracycline and ampicillin in *A. baylyi* were lower than the cut-off value of −2 for this graph. For the same reason, the data on Rhodamine 123 and acridine orange post-exposure to ampicillin in *E. coli* were not visible in this graph.

**Fig. 5.**
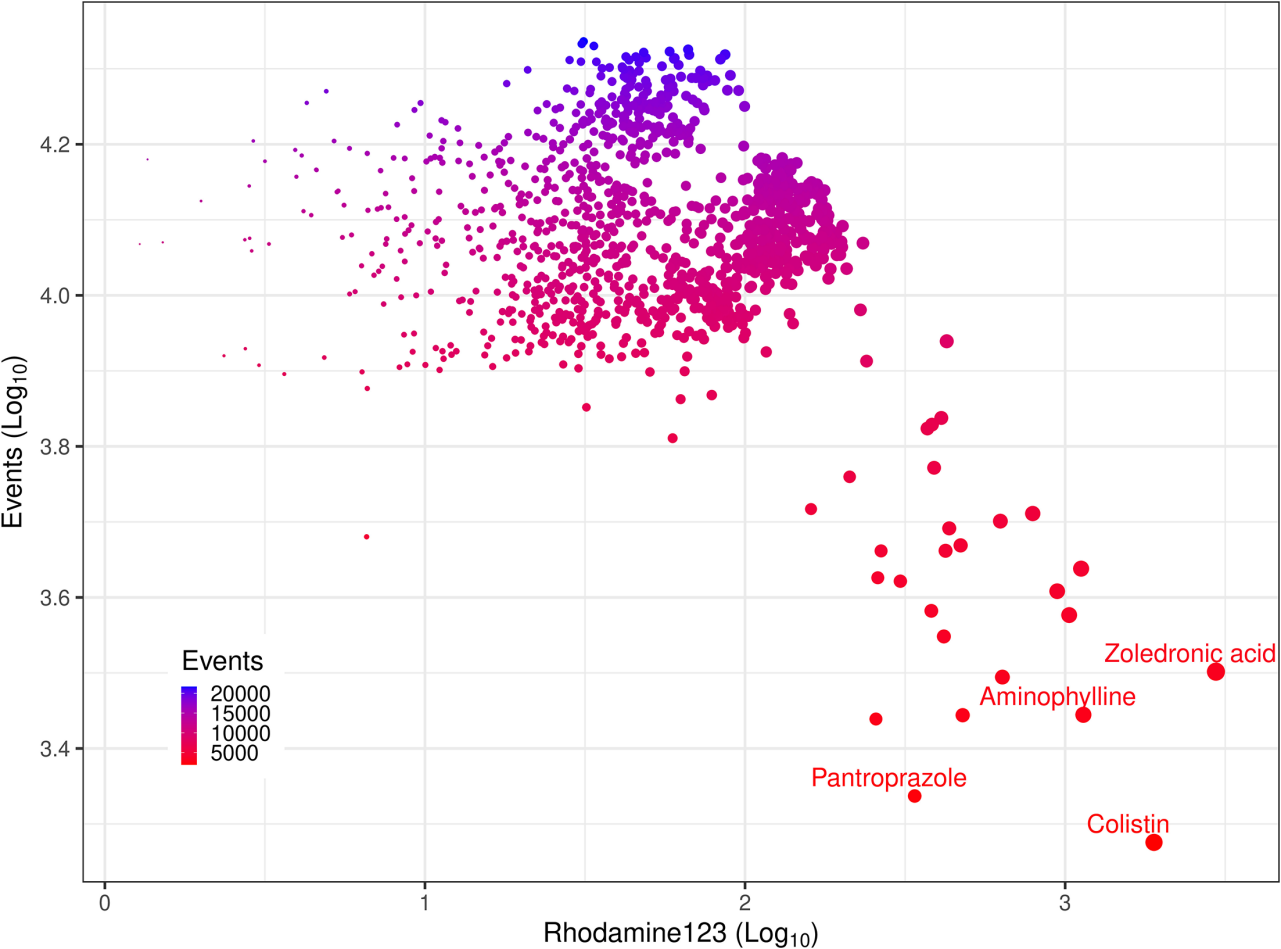
Distribution of the cytotoxic effect of a set of the Prestwick library. Flow cytometric detection of fluorophore uptake cell count [events (single cells), ordinate] and dye uptake (Rhodamine 123, abscissa) in *E. coli* exposed to 1,280 drugs from the Prestwick library. Assay conditions are detailed in Methods. Compounds with minimal toxic effect (higher number of events and low permeability to Rhodamine 123) were in the top and left areas of the plot, while high cytotoxicity, similar to that of colistin, was represented towards the lower and right section of the plot. The size of the dots increases towards the higher signals of Rhodamine 123, and the colour gradient from blue to red follows the decreasing numbers of events (cell count).

Five fluorophores were observed to accumulate in *E. coli* and *A. baylyi* at higher levels post-exposure to cell membrane-acting antibiotics such as colistin and pentamidine versus intracellularly acting antibiotics such as tetracycline (Fig. 4). The cytotoxic effect (visualized here as accumulation of fluorophores) of inhibitors of the cell wall synthesis such as ampicillin is not detectable within the time frame of these assays (i.e. 30-min incubation in antibiotics followed by 10 min incubation in fluorophore – other conditions in Methods). Cell lysis at similar time frames has been observed in *E. coli* only at approximately threefold higher concentrations of ampicillin than we used here [31]. From those fluorophores, Rhodamine 123 – to which viable cells are broadly and functionally impermeable – was the dye of choice for a proof of concept of flow cytometry screening for bactericidal compounds. We tested 1280 compounds from the Prestwick library of approved drugs (Table S3) against *E. coli*. The cytotoxicity of those drugs against *E. coli* was estimated via the accumulation of Rhodamine 123 after 30-min post-exposure to each of the Prestwick chemical compounds. We ran two biological replicates for this screen (Table S4). The trends were similar in both runs, and the data for one replicate are illustrated in Fig. 5.

Most drugs did not reduce the cell count (number of events by flow cytometry) below 7,000 events (the negative control of 1% DMSO had counts of over 20,000 events) (Fig. 5). The cellular accumulation of Rhodamine 123 on the other hand spread across two orders of magnitude (Fig. 5). Three compounds delimited the boundary of these data: aminophylline, pantoprazole sodium and zoledronic acid hydrate (Fig. 5). Pantoprazole reduced the cell count to the lowest level of ~2000 events (Fig. 5). Therefore, these compounds were interpreted to be antimicrobials as an ‘off-target’ activity.

### Pantoprazole, theophylline and zoledronic acid showed measurable growth inhibitory concentrations for Gram-negative bacteria *E. coli* and *A. baylyi*

**Fig. 6.**
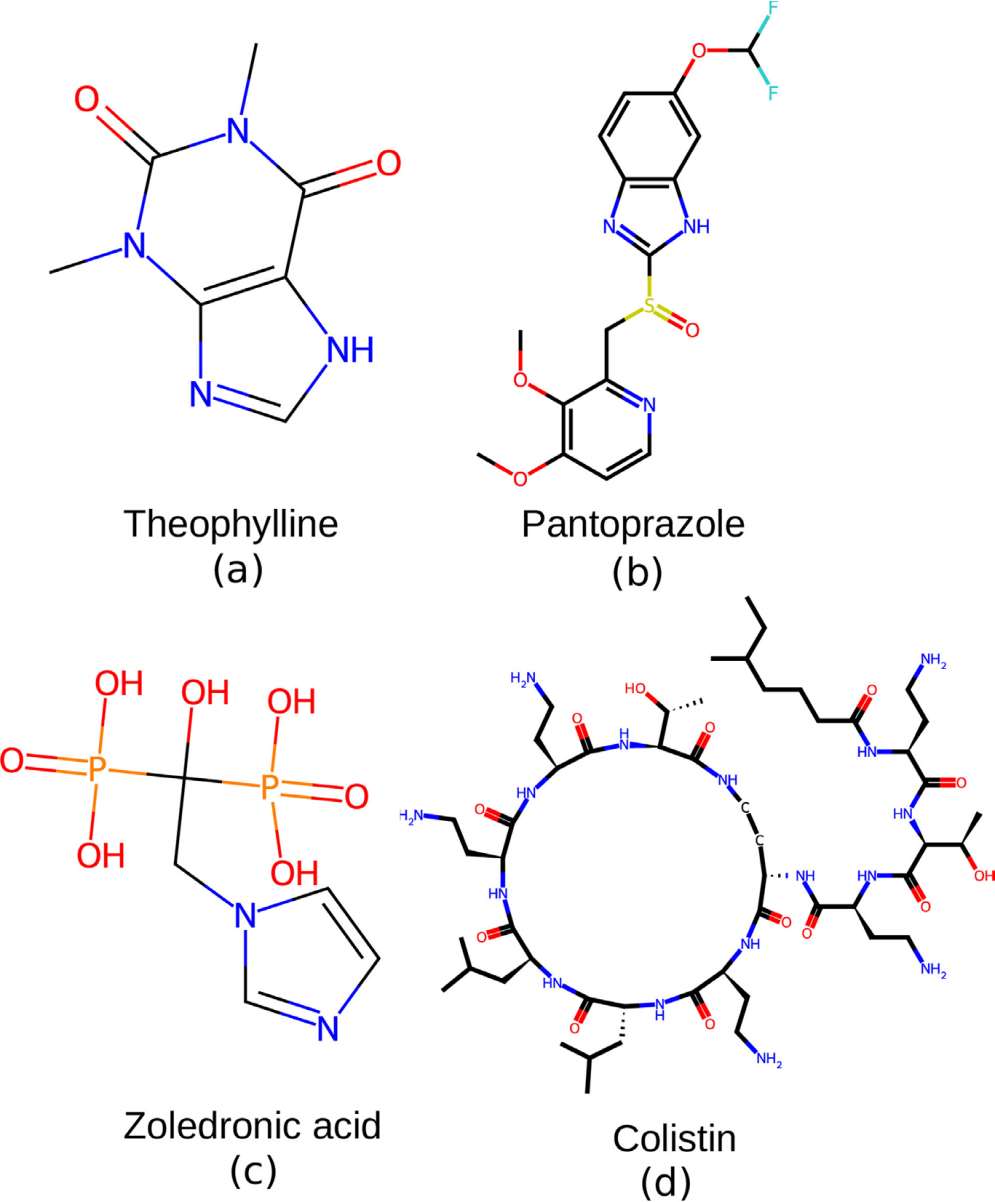
Compounds with apparent antimicrobial activity from the screen of the Prestwick library by flow cytometry. (**a**) Theophylline (MW 180), (**b**) pantoprazole (MW 383), (**c**) zoledronic acid (MW 272) and (**d**) colistin (MW 1,155). Colistin was used as the control for a known bactericidal in the growth inhibitory assays (Fig. 7).

**Fig. 7.**
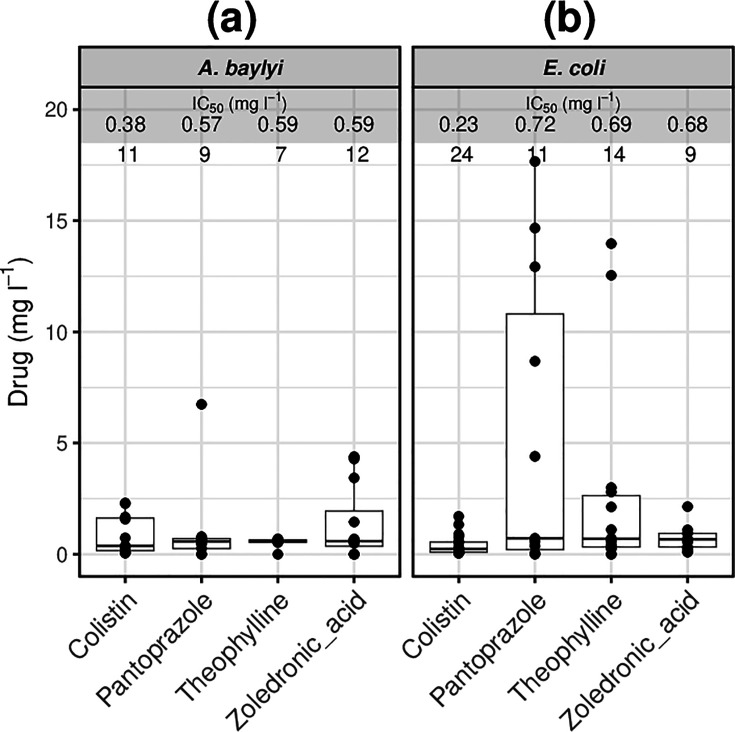
Growth inhibitory assays in *E. coli* and *A. baylyi*. (**a**) *A. baylyi.* (**b**) *E. coli*. The median values for the IC_50_ values (mg l^−1^) of colistin, pantoprazole, theophylline and zoledronic acid are presented for *A. baylyi* and *E. coli*. Data represented at least five biological replicates with four technical replicates each (black dots).

From the screening of the Prestwick library, pantoprazole, theophylline and zoledronic acid (Fig. 6) were denoted to have rapid killing properties given their effects in the reduction of number of events and fluorophore accumulation after 30 min as presented in previous sections. This timescale has been shown to be sufficient for bactericidals such as polymyxins to kill over 99% of a given inoculum [32,33]. With the inclusion of the polymyxin colistin, end-point growth inhibitory assays were then carried out for both *E. coli* and *A. baylyi* (Fig. 7) to confirm and compare their antibacterial effects. In *A. baylyi*, pantoprazole, theophylline and zoledronic acid displayed IC_50_ values in a narrow range of 0.57 to 0.59 mg l^−1^ (Fig. 7a). A similarly close range was seen for all three compounds in *E. coli* (0.68–0.72 mg l^−1^) (Fig. 7b). All IC_50_ values observed were within the same order of magnitude of that of colistin.

## Discussion

The discovery of new antibiotics is still a sporadic event measured in decades [34,36]. At the same time, infections caused by Gram-negative bacteria maintain their critical status. Carbapenem-resistant *A. baumannii*, carbapenem-resistant *Enterobacterales* and third-generation cephalosporin-resistant *Enterobacterales* top the most recent of the WHO’s Bacterial Priority Pathogens List [37]. The situation with *A. baumannii* is particularly challenging because, among other factors, of the limited antibiotic treatment options [37]. The importance of targeted high-throughput platforms for the screening of antimicrobials is self-evident, particularly for the screening of highly effective bactericides. We consider cell membrane-acting antibiotics (direct interaction with prominent components of outer and inner cell membranes) to be a key trait of bactericidal antibiotics [30,38,42]. However, the list of antibiotics of current clinical relevance with such a property is limited (Table 1).

Antibiotic discovery can be done both phenotypically and/or by choosing particular targets [43]. The former has the advantage that it can anticipate issues with cell efflux pumps [44,46]. For phenotype-based antibiotic research, there are flow cytometry and cytological workflows. The latter uses fluorescent microscopy of stained bacteria (including a membrane-impermeable reporter) [47]. This methodology has been used to create classification models of antibiotic effects [47]. However, flow cytometry continues to be a favoured approach to investigate the mechanisms of action of antibiotics due to the advantage of high-throughput, single-cell interrogation and quantitative multiparametric outputs [48].

The work presented here focused on an approach to facilitate the discovery of rapid killing and cell-permeating agents. We built on our experience in cell phenotyping using extensive numbers of fluorogenic chemicals as reported before for *E. coli* [15] and complemented here for *A. baylyi*. Together with a representation of antibiotics of clinical relevance, we provided a flow cytometry platform to identify other cell membrane-binding drugs that could be in themselves rapid acting antimicrobials. This is the basis of the paradigm of drug repurposing [49].

The flow cytometry-based screening of a sample of the Prestwick library of mostly FDA-approved drugs here delivered three candidates for compounds with anti-Gram-negative activity. Aminophylline is a mix of the active compound theophylline and ethylenediamine in a 2 : 1 ratio, used mainly for its bronchodilator effects via the interaction with several adenosine receptors, phosphodiesterases and histone deacetylases [50]. Pantoprazole is an H+, K(+)-ATPase inhibitor [51], which as part of its effects in the treatment of gastric and duodenal ulcers seems to have a direct antimicrobial action on *Helicobacter pylori* [52], as well as other bacteria [53]. Zoledronic acid is a synthetic imidazole bisphosphonate analogue of pyrophosphate with anti-bone-resorption activity. It binds to hydroxyapatite crystals in the bone matrix and also inhibits farnesyl pyrophosphate synthase, an enzyme involved in terpenoid biosynthesis [54]. Zoledronic acid also inhibits the potassium ATP-sensitive channel [55]. All three examples seemed to have the effects of being bactericidal as judged by flow cytometry parameters, as they were fast-acting and cell permeating. Equally relevant, all three examples represent chemical scaffolds that are different from known bactericidal antibiotics such as colistin.

Molecules with membrane-acting properties against bacteria can serve different purposes. For instance, polymyxins have been used to increase the *in vitro* accumulation of antibiotics with poor cellular penetration [56]. Such a permeabilization strategy would be relevant in naturally resistant Gram-negative bacteria as in the case of *Pseudomonas aeruginosa*. These bacteria have only one-eighth of the net permeability of a typical *E. coli* cell and three times the number of resistance-nodulation-division pumps [57].

## Abbreviations

PCA: principal component analysis
